# Transcriptional changes when *Myxococcus xanthus* preys on *Escherichia coli* suggest myxobacterial predators are constitutively toxic but regulate their feeding

**DOI:** 10.1099/mgen.0.000152

**Published:** 2018-01-18

**Authors:** Paul G. Livingstone, Andrew D. Millard, Martin T. Swain, David E. Whitworth

**Affiliations:** ^1^​ IBERS, Aberystwyth University, Cledwyn Building, Penglais Campus, Aberystwyth, Ceredigion, SY23 3DD, UK; ^2^​ University of Leicester, Leicester, UK

**Keywords:** antimicrobial activity, mixed culture, Myxobacteria, predatome, outer membrane vesicles, transcriptome

## Abstract

Predation is a fundamental ecological process, but within most microbial ecosystems the molecular mechanisms of predation remain poorly understood. We investigated transcriptome changes associated with the predation of *Escherichia coli* by the myxobacterium *Myxococcus xanthus* using mRNA sequencing. Exposure to pre-killed prey significantly altered expression of 1319 predator genes. However, the transcriptional response to living prey was minimal, with only 12 genes being significantly up-regulated. The genes most induced by prey presence (*kdpA* and *kdpB*, members of the *kdp* regulon) were confirmed by reverse transcriptase quantitative PCR to be regulated by osmotic shock in *M. xanthus*, suggesting indirect sensing of prey. However, the prey showed extensive transcriptome changes when co-cultured with predator, with 40 % of its genes (1534) showing significant changes in expression. Bacteriolytic *M. xanthus* culture supernatant and secreted outer membrane vesicles (OMVs) also induced changes in expression of large numbers of prey genes (598 and 461, respectively). Five metabolic pathways were significantly enriched in prey genes up-regulated on exposure to OMVs, supernatant and/or predatory cells, including those for ribosome and lipopolysaccharide production, suggesting that the prey cell wall and protein production are primary targets of the predator’s attack. Our data suggest a model of the myxobacterial predatome (genes and proteins associated with predation) in which the predator constitutively produces secretions which disable its prey whilst simultaneously generating a signal that prey is present. That signal then triggers a regulated feeding response in the predator.

## Data Summary

1. Reads from all sequencing experiments are deposited under accession numbers SRX3143879, SRX3143880, SRX3143934, SRX3143935, SRX3143947, SRX3143951, SRX3143956, SRX3143960 and SRX3143962 at the Sequence Read Archive: https://www.ncbi.nlm.nih.gov/sra.

**Impact Statement**

Predation is a fundamental ecological process, but within microbial ecosystems the mechanisms of predation remain poorly understood for most predators. We investigated transcriptome changes associated with the predation of *Escherichia coli* by the ‘wolf-pack’ myxobacterial predator *Myxococcus xanthus* using mRNA sequencing. While large-scale changes in gene expression were observed in the prey organism, exposure to prey altered the expression of only a small number of genes in the predator (osmoregulation genes). It seems that predator genes required for prey killing are expressed constitutively, and the predator instead responds to the death throes of the prey – more reminiscent of a web-building spider than a wolf-pack. This study provides a framework for further investigations into the gene regulatory changes associated with microbial predation, which will inform future efforts to exploit the antimicrobial activity of predators in the control of problem prey microbes.

## Introduction

Across the natural world, predation is a virtually ubiquitous ecological phenomenon which drives the evolution of predator and prey organisms. Operating at the cellular level, microbial predation is an arms race of chemical warfare, which has been exploited historically to produce antibiotics for the health industry [1, 2]. The soil-dwelling myxobacteria are a well-described order of predatory bacteria, which feed co-operatively on a broad range of prey organisms [3–5]. However, although myxobacterial molecular genetics has been studied for several decades, knowledge of the mechanisms involved in their predatory activity remains sparse [6].

Numerous studies have shown that myxobacteria exhibit predatory activity against a broad range of prey, including Gram-negative bacteria, Gram-positive bacteria and fungi [4, 5, 7, 8]. While the prey range of individual myxobacterial isolates is broad, it is also patchy, with a great deal of variation in the efficiency of predation against particular prey species and strains [4, 5, 9]. The broad prey range of myxobacteria is believed to be a consequence of their mode of predation, described as ‘wolf-pack’ predation [8, 10]. Myxobacteria secrete large quantities of digestive enzymes and metabolites into the extracellular commons, which can then lyse prey cells in their vicinity [11]. Nutrients co-operatively released from dead prey are then thought to be assimilated by the predators [12].

Recent studies have shown that the outer membrane vesicles (OMVs) secreted by myxobacteria are packed with a cargo of secondary metabolites and are also enriched in hydrolytic enzymes [11, 13]. OMVs and OMV-free culture supernatant can kill prey organisms [11, 14], and while antibiotics assist predation by increasing susceptibility of the prey to attack, additional factors are thought to be required for prey killing and consumption [15]. Those additional factors are potentially specific to particular predatory contexts; for instance, predation is affected by the identity of the prey organism and by the nature of the substrate on which predation occurs [4, 5].

Comparative genomics and proteomics studies have augmented our knowledge of the genes and proteins involved in predation (the predatome), hinting at possible mechanisms involved in predation [16]. However, only a handful of studies have focused on transcriptomic changes associated with predation [17], and those have dealt exclusively with the transcriptome of the predator. Several studies have looked at transcriptome changes in mixed cultures which exhibit symbiotic, cross-feeding or competitive relationships [18–20], but to our knowledge no study has yet investigated the transcriptional changes of bacterial predator and prey in a mixed culture actively engaged in predation.

With the growing phenomenon of antimicrobial resistance threatening global security, there is a desperate need for new ways to combat the problem of resistant infections. Predatory microbes such as the myxobacteria are promising candidates for natural products research, and deciphering the mechanisms by which they kill prey is important for the rational exploitation of their inherent antimicrobial activity. In this study we used transcriptome sequencing to investigate changes in gene expression in the model myxobacterium *Myxococcus xanthus* and its prey *Escherichia coli*, in a mixed culture exhibiting active predation. We hypothesized that exposure to live prey would induce genes required for prey killing, and that exposure to pre-killed cells would emulate gene expression changes associated with later phases of predation such as nutrient assimilation.

## Methods

### Growth conditions

*M. xanthus* DK1622 [21] and *E. coli* Top10 (Invitrogen) were each grown in duplicate in LBCY medium (50 % Luria-Bertani medium and 50 % DCY medium [11]) with shaking at 30 °C overnight, to give mid- to late exponential phase cultures (optical densities of ~2 at 600 nm). Cultures were sedimented by centrifugation at 10 400 ***g*** for 10 min, washed twice in TM buffer [11], split into two halves, and then resuspended in 1/10 volume of TM buffer (first half) and LBCY (second half). A portion of each *E. coli* sample was then heated to 100 °C for 1 min to kill those cells. *M. xanthus* and *E. coli* (live and dead) samples (in either LBCY or TM) were then mixed in various combinations with LBCY or TM, with or without other cells, such that the resulting cultures were of the same cell density, in either full-strength TM or LBCY. Six experimental conditions were used: *LIVE* (*M. xanthus* and live *E. coli* in TM), *DEAD* (*M. xanthus* and dead *E. coli* in TM), *Predator Starvation* (*M. xanthus* in TM), *Prey Starvation* (live *E. coli* in TM), *Predator Nutrition* (*M. xanthus* in LBCY) and *Prey Nutrition* (live *E. coli* in LBCY). Each condition was quadruplicated, with each replicate being a unique combination of *E. coli* and *M. xanthus* preparations deriving from separate initial cultures. A fifth replicate of the *LIVE* condition was generated, which was sampled hourly to determine the number of surviving *E. coli* cells.

### Generation of samples for mixed-culture transcriptomics

Liquid cultures were incubated at 30 °C from *T*=0 h (in sealed Eppendorf tubes without shaking), and until *T*=5 h a small sample was withdrawn hourly from the fifth *LIVE* condition replicate for serial dilution and spreading onto LB plates. Plates were incubated at 37 °C overnight and colonies were enumerated to give counts of viable prey. Guided by a pilot experiment, samples of each quadruplicated culture condition were harvested at *T*=4 h, cells sedimented by centrifugation, washed, and the resulting pellets stored at −80 °C prior to RNA extraction. At *T*=4 samples were also taken from each *LIVE* replicate for serial dilution and plating. After incubation overnight the density of viable prey in each replicate had reduced to 10±2 % of that at *T*=0, indicating that predation had actively occurred in those cultures, and that there were still significant numbers of surviving prey cells.

### Samples for testing culture supernatant and OMV-induced transcriptome changes

Cultures of *E. coli* Top10 were grown in LB (in quadruplicate) until they had reached mid- to late exponential phase. Cells were adjusted to an optical density of 2 at 600 nm, washed and resuspended into 1 ml of TM buffer. Then, 100 µl of cells (~8×10^9^ cells) were mixed with 500 µl of *M. xanthus* OMVs (condition *OMV*), *M. xanthus* culture supernatant (condition *Supernatant*) or TM buffer (condition *Prey Starvation 2*), and incubated at 37 °C in Eppendorf tubes, without shaking. Samples were harvested after 90 min, pelleted and washed cells were stored at −80 °C until RNA extraction. *M. xanthus* OMVs and supernatant were prepared from cultures of *M. xanthus* DK1622 as described by Evans *et al.* [11]. Protein concentration was measured using the Qubit Protein Assay Kit (Life Technologies); OMVs and supernatant had a protein concentration of 358 and 218 µg ml^−1^, respectively.

### RNA extraction and sequencing

RNA was extracted using the ‘miRNEasy mini’ kit (Qiagen 217004), removal of rRNA was undertaken using the ‘Ribo-Zero Magnetic Kit (Bacteria)’ from Epicentre (MRZB12424) and construction of cDNA libraries was performed with the ‘Truseq stranded mRNA’ library preparation kit from Illumina (RS-122-2101), all used according to the manufacturers’ instructions. Sequencing was carried out using an Illumina HiScanSQ system in the IBERS Translation Genomics facility, Aberystwyth, UK.

### RT-qPCR of *kdp* genes

Primers were designed to amplify fragments of the *kdpA* gene, the *kdpB* gene, the *kdpAB* junction and the 16S rRNA gene (as reference). Specific amplification of genomic DNA was confirmed by Sanger sequencing. Primers 16SF (CAAGGGAACTGAGAGACAGG) and 16SR (CTCTAGAGATCCACTACTTGCG) amplified a 121 bp portion of the 16S rRNA gene, primers KdpAF (TGATGAACGCCACCCTCTACG) and KdpAR (TAGGGCGCGAAGTTCTGGA) amplified a 97 bp portion of the *kdpA* gene, primers KdpBF (ATCATCATCCTGCTCATCCCC) and KdpBR (CAGCACGTCGATGACCTTGAT) amplified a 140 bp portion of the *kdpB* gene, while primers KdpABF (CCATCGTCGAGCACTTCCT) and KdpABR (GGTTTGAGCAGTGAGGCGT) amplified a 99 bp region spanning the junction of the *kdpA* and *kdpB* genes. *M. xanthus* DK1622 was grown in DCY medium for 4 days with and without 0.2 M NaCl to induce osmotic stress. Cultures were subjected to centrifugation at 10 400 ***g*** for 10 min and the resulting pellet was washed with TM buffer. RNA extraction was undertaken using a standard phenol/chloroform method [22]. RNA was quantified using a Nanodrop instrument (ND-1000). cDNA was synthesized using the Tetro cDNA Synthesis Kit (Bioline). Real-time PCR was carried out on a LightCycler480 II using the standard SYBR Green1 PCR mix (PCR Biosystems) in a 25 µl volume (23 µl PCR mix + 2 µl cDNA). The melting curves of quantitative PCR products were checked for non-specific reactions, and the fold changes of *kdpA*, *kdpB* and *kdpAB* in the test sample were compared with that of the reference 16S rRNA gene. Relative quantification of target gene expression was calculated using the 2ΔΔCt method.

### Transcriptome mapping

There were 16 *E. coli* and 16 *M. xanthus* sets of reads, corresponding to four replicates for each of the four experimental conditions. Trimmomatic was used to clean the reads before downstream processing. This involved removing adaptor sequences, leading and trailing bases with quality thresholds below 20, sliding window trimming (with parameters 4 : 15), and removing reads less than 36 bp in length. After cleaning, the remaining paired reads were mapped to the respective genomes to calculate expression values. This was performed with the RSEM software, using the ‘rsem-calculate-expression’ command with bowtie2 and the following options to estimate the read start position distributions (‘- -estimate-rspd’) and to add the gene or transcript name to the output (‘- -append-names’). The genomes used were *M. xanthus* DK1622 strain (accession: CP000113.1) and the *E. coli* K12 substrain MG1655 (accession: NC_000913.3). The output from RSEM included FPKM (fragments per kilobase of transcript per million mapped reads).

### Differential expression analysis

The EdgeR Bioconductor package [23] was used to analyse differently expressed genes and to undertake hierarchical clustering of the data. Significantly up-regulated and down-regulated genes were defined using a false discovery rate of less than 0.001, a *P*-value of <0.05 and a minimum 1 log_2_(fold)-change of gene expression (*Low Stringency*). Further filtering focused attention on the genes whose (log_2_(fold)) expression changed by >2 (*High Stringency*). Gene Ontology (GO) annotations and analyses of up/down-regulation of pathways were carried out using the DAVID online tool [24, 25]. Circos diagrams were constructed using CircosVCF [26], plotting differential expression for each coding sequence. Also included were genomic islands as predicted by Island Viewer 3.0 [27], secondary metabolite gene clusters as predicted by antiSMASH 4.0 [28], and signalling proteins as predicted by P2CS and P2TF [29, 30].

### Supporting data

Reads from all sequencing experiments are deposited under accession numbers SRX3143879, SRX3143880, SRX3143934, SRX3143935, SRX3143947, SRX3143951, SRX3143956, SRX3143960 and SRX3143962 at the Sequence Read Archive (Data Citation 1).

## Results

### Transcriptome sequences of predator and prey during predation

**Fig. 1.**
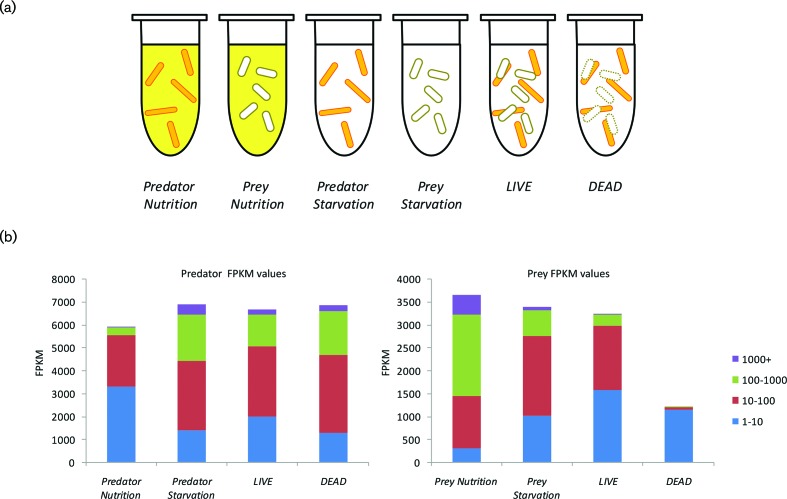
Gene expression under the six experimental conditions. (a) Experimental conditions for predator–prey RNAseq. *M. xanthus* predator (orange rods) and *E. coli* prey (white rods) were incubated separately or together, in either buffer (white background) or LBCY nutrient medium (yellow background). *E. coli* cells added to condition *DEAD* were heat-killed beforehand. (b) Gene expression (FPKM values) of the 7400 genes of the *M. xanthus* predator (left four columns) and the *E. coli* prey (right four columns), binned and coloured by magnitude, for each of the experimental conditions.

To identify conditions in which mixed populations of *M. xanthus* and *E. coli* would be homogenous and actively engaged in predation, a growth medium (LBCY) was developed which supported the growth of each species at a similar growth rate to that exhibited in their standard growth media (DCY and LB, respectively). Dense cultures of both species were washed and cells were introduced at high density into nutrient (LBCY) or nutrient-free (TM buffer) media, either individually or in mixed culture. A portion of *E. coli* cells was heat-killed to give dead prey cells prior to sub-culturing. Six experimental conditions were used (Fig. 1): *LIVE* (*M. xanthus* and live *E. coli* in TM), *DEAD* (*M. xanthus* and dead *E. coli* in TM), *Predator Starvation* (*M. xanthus* in TM), *Prey Starvation* (live *E. coli* in TM), *Predator Nutrition* (*M. xanthus* in LBCY) and *Prey Nutrition* (live *E. coli* in LBCY). Each condition was biologically quadruplicated.

To emulate as closely as possible the natural conditions of predation on a surface, whilst limiting the heterogeneity of the resulting populations, the high-cell-density liquid cultures were incubated without shaking, allowing cells to sediment. After 4 h (with 10±2 % prey survival), cultures were harvested for RNA extraction, ribo-depletion, cDNA synthesis and DNA sequencing. Reads were mapped to the genomes of *M. xanthus* and *E. coli* and an FPKM value was assigned to each gene reflecting its relative expression level. FPKM is a within-sample normalized transcript expression measure that is independent of library-size effects. Reads mapping to RNA genes were excluded, and significant changes in relative expression level between conditions were identified using EdgeR. Differentially expressed genes were assigned GO terms and KEGG pathway membership using the DAVID online tool, which also identified pathways and GO terms significantly enriched for differentially expressed genes.

One replicate for the *Predator Nutrition* condition failed to generate sequence data, and the *DEAD* condition gave only 4 % of reads mapping to *E. coli*, with a mean sum of FPKM values of just 5760. However, the other samples gave a mean sum of FPKM values of 1.4 million per replicate, spread over 7400 *M. xanthus* genes, and a mean sum of FPKM values of 1.2 million per replicate, spread over 3863 *E. coli* genes (FPKM values for each gene in each replicate are provided as File S1, available in the online version of this article). Fig. 1 shows the distribution of FPKM values for the predator and prey transcriptomes under each experimental condition. Multi-dimensional scaling (MDS) plots of the datasets show non-overlapping clustering of replicates by experimental condition (File S2). For the *M. xanthus* transcriptomes, the *Predator Nutrition* replicates clustered noticeably apart from the other conditions, with the *DEAD* transcriptome being the most similar condition. Clusters for the *LIVE* and *Predator Starvation* conditions were discrete but were closer together than any other pair of conditions. For the *E. coli* transcriptomes, replicates of the three conditions *LIVE*, *Prey Nutrition* and *Prey Starvation* clustered separately, with the *Prey Starvation* furthermost from the other clusters (File S2).

The proteomes of *M. xanthus* cell extracts, culture supernatant and OMVs have been characterized quantitatively previously [13]. The relative abundance of proteome proteins was compared with the FPKM values of the encoding genes under *Predator Starvation* and *Predator Nutrition* conditions, by calculating the Pearson product moment correlation coefficient, *r*. For each proteome, there was a significant (*P*<0.00001) positive correlation between proteome proportion and the FPKM values of genes encoding proteins from both conditions, although only the cell extract proteome gave correlations with *r*^2^ values of >0.25 (strong association). The correlation with the largest *r*^2^ value (0.3833) was between the cell extract proteome and the *Predator Starvation* FPKM values, perhaps unsurprisingly, as cell extracts were generated from cultures that had been pre-incubated in TM buffer [13].

### *M. xanthus* does not perceive live prey as food

**Fig. 2.**
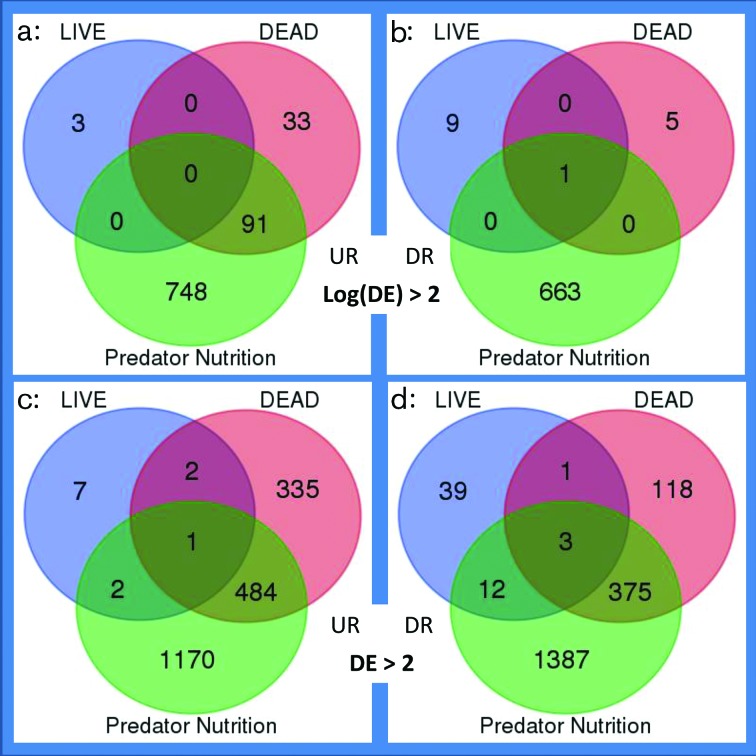
*M. xanthus* genes exhibiting differential expression (DE) on exposure to nutrients (*Predator Nutrition*), prey (*LIVE*) and pre-killed prey (*DEAD*), when compared with a nutrient-free control condition (*Predator Starvation*). DE gene numbers are shown for both high (a, b) and low (c, d) stringency filtering criteria [log(DE)>2 and DE>2, respectively]. Up-regulated genes (UR) are shown on the left (a, c) and down-regulated (DR) genes on the right (b, d).

**Fig. 3.**
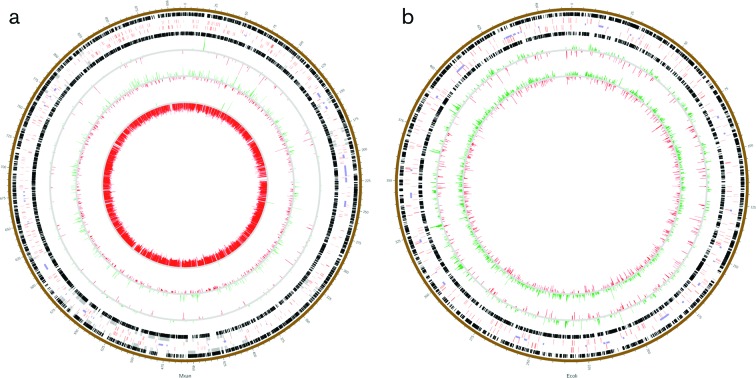
Circos diagrams of the *M. xanthus* (a) and *E. coli* (b) genomes. Numbers around the diagrams each designate 10 000 bp. Black boxes represent genes encoded on the two DNA strands, red boxes designate genes encoding signalling and/or DNA-binding proteins, purple boxes are genomic islands, and grey boxes denote genes of secondary metabolite biosynthesis clusters. Nested rings represent genes differentially expressed (up-regulated in green and down-regulated in red) when comparing transcriptome datasets. The three comparisons in (a) are *LIVE* versus *Predator Starvation* (outermost), *DEAD* versus *Predator Starvation*, and *Predator Nutrition* versus *Predator Starvation* (innermost). The two comparisons in (b) are *LIVE* versus *Prey Starvation* and *Prey Nutrition* versus *Prey Starvation*.

Differences in relative gene expression between experimental conditions were assessed, and genes defined as up-regulated (UR) or down-regulated (DR) according to low- and high-stringency criteria [differential expression (DE)>2 and log(DE)>2, respectively]. To simplify analysis, all transcriptomes were benchmarked for comparison against the *Predator Starvation* condition and lists of UR/DR genes are provided as File S3 and illustrated in Fig. 2. The greatest number of UR/DR genes was identified when comparing the *Predator Nutrition* and *Predator Starvation* conditions (3434/1503 at low/high stringency, respectively), while the lowest number of UR/DR genes was identified in the *LIVE* versus *Predator Starvation* comparison (67/13 at low/high stringency, respectively). Fig. 3(a) shows the distribution of differentially expressed genes around the *M. xanthus* genome.

With high-stringency filtering, very few genes were found to be UR/DR in more than one condition, with the exception of one gene (MXAN_1508, a hypothetical protein) which was DR in all three conditions, and 91 genes found to be UR in both the *Predator Nutrition* and the *DEAD* conditions. Under low-stringency filtering there remained relatively few genes UR/DR in multiple comparisons (880), of which 98 % (859) were UR/DR in both the *DEAD* and the *Predator Nutrition* conditions.

The low number of genes UR/DR in *LIVE* compared to *Predator Starvation*, and the large overlap between responses to *DEAD* and *Predator Nutrition* conditions, suggests that *M. xanthus* responds only minimally to the presence of living prey under these experimental conditions, although it responds to pre-killed prey similarly (albeit differently) to if it had been provided with nutrients in the form of rich medium.

### Live prey induce an osmotic stress response while dead prey induce sugar metabolism genes

Using the more stringent filtering criterion [log(DE)>2], very few genes were found to be differentially regulated by the presence of live prey (three UR and ten DR). All three UR genes were found within a single putative operon, and included the *kdpA* and *kdpB* genes, which are part of the *kdp* (potassium-dependent) system involved in responding to osmotic stress [31]. To test whether the *kdpAB* operon of *M. xanthus* responds to osmolarity, reverse transcriptase quantitative PCR (RT-qPCR) was used to assay gene expression in the presence and absence of osmotic stress (0.2 M NaCl). RNA regions within the *kdpA* gene, within the *kdpB* gene and spanning the *kdpA-B* junction were all found to be highly induced by osmotic stress, compared to a 16S rRNA gene reference [with log_2_(fold change) values of 8.7, 10.3 and 9.9, respectively]. These data suggest that *kdpA* and *kdpB* are expressed together within an operon, and that operon is highly induced by osmotic stress. Thus, it is likely that the *kdp* operon of *M. xanthus* is induced indirectly by the presence of prey, through changes in osmolarity caused by prey presence, rather than as a direct response to the prey per se.

No obvious functional links were apparent between the DR proteins, which included a nitroreductase, a serine/threonine protein phosphatase, DNA polymerase IV, a major facilitator family transporter and a phosphotransfer system (PTS) component. With less stringent filtering, 68 predator genes were identified as differentially regulated with live prey (56 DR and 12 UP), still very few compared to the 532 and 798 genes DR and UR, respectively, in response to the presence of dead prey (Fig. 2).

The DAVID algorithm was used to investigate whether any KEGG pathways [32] were significantly UR or DR in response to *LIVE* or *DEAD* prey, based on their constituent genes meeting the high-stringency filtering criterion (Table 1). The only pathways affected by *LIVE* prey were two-component systems (genes *kdpA* and *kdpB* were UR, and are under the control of the KdpDE two-component regulatory system), but *DEAD* conditions up-regulated the PTS, amino sugar and nucleotide sugar metabolism, and fructose and mannose metabolism pathways, which are all involved in sugar uptake and metabolism. The same three pathways were also UR in the *Predator Nutrition* condition (Table 1), alongside the base excision repair pathway (and with the ribosome KEGG pathway being DR). The overlap between the responses to *DEAD* and *Predator Nutrition* conditions reinforces the idea that *M. xanthus* perceives dead prey cells as food, although responses to the two conditions are different. Of the 1541 genes UR or DR in either condition (with high-stringency filtering), only 92 (6 %) are co-ordinately regulated in both conditions (Fig. 2).

**Table 1.** Pathways of *M. xanthus* differentially regulated in different experimental conditions (italicized) compared to the *Predator Starvation* experimental condition

Down-regulated	Up-regulated
*LIVE*	
Nil	mxa02020 : Two-component system
*DEAD*	
Nil	mxa00051 : Fructose and mannose metabolism
	mxa00520 : Amino sugar and nucleotide sugar metabolism
	mxa02060 : Phosphotransferase system (PTS)
*Predator Nutrition*	
mxa03010 : Ribosome	mxa00051 : Fructose and mannose metabolism
	mxa00520 : Amino sugar and nucleotide sugar metabolism
	mxa02060 : Phosphotransferase system (PTS)
	mxa03410 : Base excision repair

### Prey gene expression responds dramatically to the presence of predator

The expression of *E. coli* genes when mixed with predator under *LIVE* conditions was compared to expression under *Prey Starvation* conditions. At high stringency, 115 genes were DR and 521 genes were UR, while with low-stringency filtering, 441 genes were DR and 1093 were UR (File S3). This is a remarkably large number of genes when compared to the myxobacterial response to the same conditions, which involved only 67 differentially regulated genes under low-stringency filtering. Fig. 3(b) shows the distribution of differentially expressed genes around the *E. coli* genome.

Querying DAVID with the high-stringency gene lists, 15 pathways were found to be significantly UR (Table 2) while only one was DR (glycerophospholipid metabolism). The UR pathways involved those related to the production of antibiotics and secondary metabolites, energy and carbon metabolism, vitamin and amino acid (A, D and G) metabolism, and ribosome production (Table 2). This suggests that the *E. coli* response to predatory attack involves counter-attack, increased energy generation and protein production. The large number of differentially expressed genes precludes the identification of particular molecular targets of myxobacterial predation. Coupled with the low numbers of differentially expressed predator genes, these observations imply that the myxobacterial attack on *E. coli* is constitutive, and either is multi-factorial or triggers a highly pleiotropic response in prey.

**Table 2.** Pathways of *E. coli* differentially regulated when exposed to *M. xanthus* compared to the *Prey Starvation* experimental condition

Down-regulated	Up-regulated
*LIVE*	
eco00564 : Glycerophospholipid metabolism	eco00020: Citrate cycle (TCA cycle)
	eco00190 : Oxidative phosphorylation
	eco00250 : Alanine, aspartate and glutamate metabolism
	eco00620 : Pyruvate metabolism
	eco00640 : Propanoate metabolism
	eco00650 : Butanoate metabolism
	eco00680 : Methane metabolism
	eco00730 : Thiamine metabolism
	eco01100 : Metabolic pathways
	eco01110 : Biosynthesis of secondary metabolites
	eco01120 : Microbial metabolism in diverse environments
	eco01130 : Biosynthesis of antibiotics
	eco01200 : Carbon metabolism
	eco03010 : Ribosome
	eco04122 : Sulfur relay system

### Prey response to OMV attack is more focused than when attacked by the whole predator

The OMVs and soluble supernatant produced by cultures of *M. xanthus* are able to kill *E. coli*, and contain simple proteomes compared to whole cells [11, 13]. Twenty-three of the 75 proteins identified in *M. xanthus* OMVs were differentially regulated in the conditions tested above – all 23 were DR in the *Predator Nutrition* versus *Predator Starvation* comparison, agreeing with earlier observations that OMV production is induced by starvation [33]. However, the DR genes were relatively minor components of the OMVs, together representing only 18 % of the cargo proteome, suggesting the most abundant OMV proteins are expressed constitutively.

To test whether the prey response to OMVs and/or supernatant was more specific than that to whole predator cells, a second transcriptome sequencing experiment was undertaken (with a repeat of the *Prey Starvation* condition as control). Samples gave mean sums of FPKM values of 0.99 million per replicate, spread over 4476 *E. coli* genes (File S1). MDS plots of the datasets showed clustering of datasets by experimental condition, but with the *Supernatant* and *OMV* condition clusters overlapping (File S2).

**Fig. 4.**
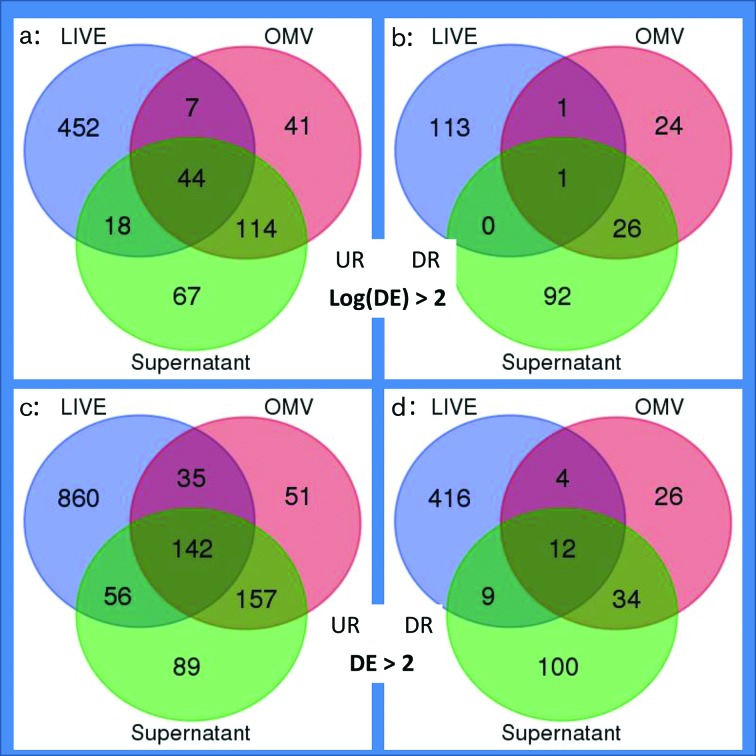
*E. coli* genes exhibiting differential expression (DE) on exposure to *M. xanthus* cells (*LIVE*), OMVs and culture supernatant (when compared with the nutrient-free control condition *Prey Starvation*) with high (a, b) and low (c, d) stringency filtering criteria [log(DE)>2 and DE>2, respectively]. Up-regulated genes (UR) are shown on the left (a, c) and down-regulated (DR) genes on the right (b, d).

Transcriptional responses under conditions *SN* and *OMV* were compared to that of *Prey Starvation 2* (prey cells in TM buffer), to identify genes specifically repressed or induced on exposure to *M. xanthus* secretions, and the resulting lists were then compared to the genes DE on exposure to predator cells (from the earlier *LIVE* versus *Prey Starvation* comparison). Fig. 4 (and File S3) shows the numbers of genes DE in the prey when mixed with predator cells (*LIVE*), OMVs (*OMV*) or culture supernatant (*Supernatant*). The number of genes DE in response to OMVs and/or supernatant was 32 % fewer than with live predator. In total, 79 % of those genes were DE in only one condition, and of the 211 which were DE in multiple conditions, 88 % were common to the *OMV* and *Supernatant* conditions. This is perhaps unsurprising, as culture supernatant composition appears to be largely a product of OMV lysis [13].

Of all the pathways induced in *E. coli* by *M. xanthus* (Table 2), only eco03010 : Ribosome was also UR in both *OMV* and *Supernatant* conditions, although a further ten pathways were found to be DE in both *OMV* and *SN* conditions (Table 3). Nevertheless, analysis of the 44 genes UR in all three conditions (*OMV*, *Supernatant* and *LIVE*) showed enrichment in five pathways, namely eco03010 : Ribosome, eco02060 : Phosphotransferase system (PTS), eco00052 : Galactose metabolism, eco00260 : Glycine, serine and threonine metabolism, and eco00540 : Lipopolysaccharide biosynthesis. These five pathways potentially highlight the physiological processes or molecular targets which *M. xanthus* primarily attacks during predation.

**Table 3.** Pathways of *E. coli* differentially regulated when exposed to both M*. xanthus OMV*s and culture *Supernatant* compared to the *Prey Starvation 2* experimental condition

Down-regulated	Up-regulated
*OMV* and *Supernatant*	
eco02010 : ABC transporters	eco00010 : Glycolysis/gluconeogenesis
eco02020 : Two-component system	eco00052 : Galactose metabolism
	eco00230 : Purine metabolism
	eco00240 : Pyrimidine metabolism
	eco00260 : Glycine, serine and threonine metabolism
	eco00521 : Streptomycin biosynthesis
	eco02010 : ABC transporters
	eco02060 : Phosphotransferase system (PTS)
	eco03010 : Ribosome

## Discussion

### Changes in gene expression during predation

The evolutionary arms race between predator and prey has selected for specialized physical and behavioural adaptations across the tree of life. While such adaptations can be easily observed and understood in metazoans, microbial predation mechanisms and the evasion strategies of prey are less apparent, and at the cellular level are likely to manifest as changes in the expression patterns of predation-associated genes. Although gene expression associated with predation has been studied previously [17, 34], here we used transcriptome sequencing to investigate predatory gene expression from the perspective of both predator and prey simultaneously.

The presence of the predator *M. xanthus* induced large-scale gene expression changes in *E. coli* prey, with hundreds of genes being UR and DR. However, the presence of live *E. coli* caused changes in the expression of only a small number of genes in *M. xanthus*. This suggests that *M. xanthus* secretes predatory molecules constitutively, independently of *E. coli* presence – as observed with its secretion of predatory OMVs. The small number of genes regulated in response to *E. coli* was surprising, however, as myxobacteria are well known for their extensive repertoire of signalling pathways, are known to respond to molecules secreted by prey, and signalling has been observed during predation [35–37]. In *Bdellovibrio*, a predatory relative of the myxobacteria, killing and consumption of prey involves widescale gene expression changes [17]. However, the apparent disparity with myxobacterial predation may be due to the different predatory strategies of *Bdellovibrio* and myxobacteria. *Bdellovibrio* invades the periplasm of a prey cell and kills the host from within, while myxobacterial wolf-pack predation involves secretion of toxins into the public commons [8, 38].

Nevertheless, the provision of dead *E. coli* did result in the DE of hundreds of genes in *M. xanthus*, primarily those involved in sugar uptake and metabolism. It would seem that *M. xanthus* predation is characterized by the exhibition of obligate toxicity towards neighbouring prey and then regulated scavenging behaviour on their death.

### The predator’s response to live prey

Consistent with constitutive expression of toxic/lytic factors, the three genes induced most by the presence of live prey were not obviously toxins or hydrolases. Rather, they belonged to the *kdp* operon, which mediates responses to changes in osmolarity [31]. The *kdp* operon encodes the KdpFABC ATPase complex, which plays a key role in potassium ion translocation during osmotic stress or potassium depletion [31].

Although studies have investigated osmoregulatory genes in *M. xanthus* [39], there have been no reports on the *kdp* genes. RT-qPCR showed that increasing the osmolarity of the surrounding medium induces expression of *kdpAB* in *M. xanthus*, suggesting that these genes are induced indirectly by the presence of prey, through increased osmolarity of the surrounding milieu. Published links between osmolarity and bacterial predation are absent from the literature, but potassium transport affects susceptibility to aminoglycosides, for example in the prey organism *Staphylococcus aureus* [40].

Expression of the Kdp system is regulated by the well-characterized *kdpDE* two-component system (TCS) signalling pathway. The myxobacteria possess extremely large complements of TCS genes [36, 41], which have been implicated in many of their behaviours. The *M. xanthus* DK1622 genome in particular encodes nearly 300 such signalling genes, of which around one-third have gene regulatory DNA-binding outputs. Therefore, it is extremely surprising that there was no evidence of more extensive TCS signalling and gene regulation during predation.

### Assimilation of prey biomass

In addition to killing prey, successful predators must also be able to degrade and incorporate the material of dead prey into progeny. In total, 130 genes were DE (at high stringency) on exposure to *DEAD* prey (Fig. 2). Three pathways were significantly UR in the presence of dead *E. coli*, although the same three UR genes were responsible in all three cases (MXAN_6532, MXAN_6533 and MXAN_6534) – all three being components of the PTS phosphotransferase sugar-uptake system. A further 30 of those 130 genes were associated with significantly enriched GO terms, COG, SMART, PIR or Interpro categories, when analysed using DAVID [25], including further PTS genes and genes involved in DNA replication, transcription regulation and glycoside hydrolysis (File S4). However, of the 130 genes DE in the presence of dead prey, the remaining 97 (74.6 %) were not associated with significantly DE metabolic pathways or enriched functional ontology terms. Of those genes, two-thirds (65) were hypothetical proteins, i.e. proteins of potentially novel function. Nevertheless, the annotations of the minority of regulated genes suggest that the predator is engaged in saccharide degradation, regulating changes in gene expression and replicating (File S4).

The functional analysis tool DAVID allows clustering of functional ontology terms into cognate clusters, providing an objective summary of functions enriched in particular gene sets [25]. File S5 provides the DAVID clusters of ontological terms enriched in the *M. xanthus* gene sets identified as being significantly DE between experimental conditions [log(DE)>2]. No clustering was found for the *LIVE* comparison (either UR or DR), or for DR genes in the *DEAD* comparison. Six clusters of enriched terms were identified amongst the UR genes in *DEAD*, while 43 clusters were identified in UR genes and 32 clusters in DR genes, for the *Predator Nutrition* comparison.

The relatively minor response to *DEAD*, compared to the extensive response to *Predator Nutrition* conditions, implies that starved *M. xanthus* are able to exploit the nutrients in dead *E. coli*, with relatively minimal changes in gene expression, although assimilation of the nutrients in rich medium requires extensive changes in gene expression. Considering terms relating to intermediary metabolism, the *DEAD* condition stimulated just sugar uptake and nucleotide binding, while the *Predator Nutrition* condition caused changes in sugar, amino acid, phospholipid, vitamin, porphyrin, peptidoglycan, isoprenoid and lipid metabolism (File S5), perhaps reflecting that the various nutrients in rich media are not as ‘well-balanced’ as those found in dead prey.

Amino acids are thought to be the preferred nutrient sources of myxobacteria [12, 42], but the pattern of transcriptome changes suggests that when growing on dead *E. coli*, sugars are metabolized more than amino acids. Perhaps sugar metabolism provides *M. xanthus* with energy, while *E. coli* amino acids are not metabolized, instead being incorporated directly into *M. xanthus* proteins.

### Prey responses to attack

While the presence of live *E. coli* had very little effect on *M. xanthus* gene expression, the presence of the predator caused widespread changes in gene expression within *E. coli* (Table 2; File S3). So many changes in gene expression were observed that it proved impossible to discriminate between genes likely to be indirectly or directly affected by *M. xanthus* attack, as opposed to those induced merely by the presence of other cells. For instance, osmotic response genes were induced in *M. xanthus* by the presence of *E. coli*, and the *kdpBCDE* genes were also induced in *E. coli* on exposure to *M. xanthus*. However, the *E. coli* response to *M. xanthus* OMVs and culture supernatant was much reduced compared to that of *M. xanthus* cells, with supernatant and OMV inducing expression of 243 and 206 genes, respectively, compared to the 521 induced by *LIVE M. xanthus* (Fig. 4).

The 44 prey genes induced in response to all three challenges showed enrichment of five pathways, including ribosome and lipopolysaccharide (LPS) biosynthesis. This may suggest that the LPS and ribosomes are direct targets of the predator’s attack. LPS is an essential component of the outer surface of Gram-negative bacteria, and contributes to the maintenance of the impermeability of the outer membrane. LPS is a molecular target for various antibacterial drugs and toxic proteins, such as those of the innate immune system [43]. The ribosome is also the target for numerous antibiotic substances [44], and it is highly plausible that myxobacteria would secrete both LPS and ribosome-targeting metabolites.

The antibiotic myxovirescin produced by *M. xanthus* DK1622 is known to inhibit *E. coli* type II signal peptidase *lspA* [45], although in our experiments we saw no differential regulation of the *lspA* gene in prey, nor of the myxovirescin biosynthesis genes. The extended myxovirescin gene cluster genes (MXAN_3926 to MXAN_3953) exhibited very low expression levels under every condition tested, and it therefore seems likely that the predatory activity in our experiments was myxovirescin-independent, with other metabolites having a bacteriocidal/bacteriostatic effect on *E. coli* [15]. Candidate metabolites include the myxalamides, which are inhibitors of the electron transport chain [46]. Intriguingly one of the pathways UR by *E. coli* on exposure to *LIVE* predator and *OMV*s was eco00190 : Oxidative phosphorylation, a pathway which includes the electron transport chain.

### Temporal and spatial aspects of predation

The transcriptomic experiment described above was designed with the expectation that exposure to live prey would induce genes required for prey killing, and that exposure to pre-killed cells would emulate gene expression changes associated with later phases of predation such as nutrient assimilation. The paucity of ‘killing’ genes identified suggests that there may be no temporal ‘programme’ as such during predation, and we suspect that a timecourse experiment would probably show the same genes DE as we observed with pre-killed prey. By making that statement, however, we are assuming that heat-killed cells are a good proxy for prey killed ‘naturally’ by the myxobacterial predatome. It could also be expected that the genes induced by dead *E. coli* but not by nutrient-rich medium would be those genes with specific roles in digesting killed prey. Of those genes, 88 % encoded hypothetical proteins, which demands further investigation of their potential roles as novel digestive/antimicrobial proteins.

Another limitation of our experimental set-up is that it was performed in liquid phase, albeit with no shaking to allow cells to sediment and form a ‘pseudo-biofilm’. Myxobacteria are surface-dwelling organisms that form spatially structured colonies, with each constituent cell having a different microenvironment and life history. Such heterogeneity is problematic for distinguishing between signal and noise in gene expression studies, so we tried to generate as homogeneous a population as possible. As predation is potentially contact-mediated [47], it is possible that cells at the vanguard of the advancing predatory colony might have different patterns of gene expression from the bulk of the colony, which our experiment would have been unable to assess.

Although our approach was designed to reduce predatory heterogeneity from a colony-wide scale to a cellular scale, most cells in our assays would not have been resident on a solid substrate during predation, as they would have been in their natural context. The nature of the substrate has a profound effect on parameters of predation, as does the identity of the prey organism [48, 49]. It is possible that our approach will have therefore missed genes that are important predatome components on specific media, or when consuming particular prey. However, we were interested in identifying ‘general’ predatome components, those that would be involved in prey killing/assimilation regardless of prey or substrate specifics. Our choice of *M. xanthus* and *E. coli* as predator and prey organisms was driven by the volume of literature describing their biology. DK1622 and Top10 are both lab-acclimatized strains and the ecological relevance of our findings is therefore unclear. In fact, DK1622 appears to be a particularly poor predator when compared to wild isolates [5]. Nevertheless, predation was observed in our assays and therefore we can be confident that mechanistically important predatome components were active during this study.

### The myxobacterial predatome

**Fig. 5.**
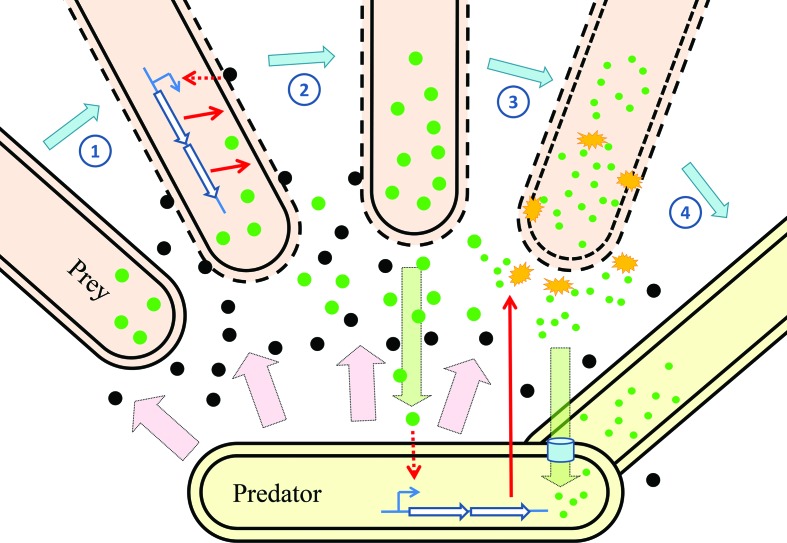
A model of transcriptional changes during predation of *E. coli* by *M. xanthus*. From left to right: prey cells close to the predator encounter its secretions such as OMVs (black circles). The predatory secretions attack the surface of the prey cell inducing changes in gene expression as the prey resist attack (1). Damage to the prey releases material (green circles) into the commons (2), which is sensed by the predator. The predator induces expression of genes for biomass assimilation, including hydrolases (yellow starbursts) which break down prey-derived material (3) for uptake by the predator (4). Pink arrows indicate constitutive secretion by the predator, green arrows denote the flow of prey-derived material, blue open arrows represent genes, while red arrows indicate the induction of gene expression and transport of gene products.

In Fig. 5 we propose a model of prey killing by *M. xanthus*. The predator secretes toxins which start to permeabilize any prey encountered, which in turn induces expression of prey genes as part of attempts to repair the cellular damage and resist the effects of the toxins. Material released from the besieged prey cell is then perceived by the predator as nutritious, prompting it to release digestive proteins for degradation and assimilation, which potentially also contribute to the demise of the prey cell.

The efficient assimilation of biomass is thought to be a prerequisite for bacterial predation, which otherwise is merely the poisoning of competitors [15], and it is unlikely that killing and assimilation are discrete processes (temporally or spatially) *in situ*. The involvement of more predators (either through reproduction or recruitment) might result in the balance of damage/repair tipping in the direction of prey death more quickly, resulting in the manifestation of myxobacterial predation as a co-operative process – more efficient when more cells are involved.

### Myxobacteria behave more like spiders than wolves

This study not only provides the first view of transcriptome-wide changes during myxobacterial predation, but also makes available datasets which give an absolute view of the composition of the myxobacterial transcriptome; previous whole transcriptome studies on myxobacteria have used microarrays to characterize relative changes in gene expression between two conditions. We expect the datasets for conditions *Predator Nutrition* and *Predator Starvation* to be particularly useful to the myxobacterial field, given the importance of nutrient sensing and starvation to the initiation of multicellular fruiting body formation [50]. We also believe this is the first description of the transcriptional changes in both a bacterial predator and its prey bacterium responding to each other’s presence in co-culture.

This study demonstrated large-scale changes in gene expression when *M. xanthus* was exposed to pre-killed *E. coli*, although a similarly large-scale response to live *E. coli* was not observed, implying that prey-killing is essentially constitutive. This scenario fits with a mechanism of OMV-mediated killing, as OMV production is constitutive, whereas production of secondary metabolites is often regulated. Conversely, the *E. coli* response to predation implies that the cell surface (LPS) and ribosomes are under direct attack, in turn suggesting the action of OMVs and secondary metabolites, respectively. Further studies are warranted, to provide mechanistic insights into the genetic basis of predation and prey susceptibility, potentially paving the way for the development of novel antimicrobial therapies. To this end we are currently investigating other myxobacterial predators, which exhibit altered prey ranges from the type strain DK1622, and testing individual OMV components for antimicrobial activity.

Myxobacterial predation is often likened to ‘wolf-pack’ predation, based on suggestions that high cell densities are required for predation [8], but our data suggest that web-building spiders may provide a better analogy [51]. With both spiders and myxobacteria, the predator secretes an external structure which disables prey whilst generating a signal that prey is present. That signal then triggers a response in the predator to consume the disabled prey. Thus, the predator only responds indirectly to prey, sensing merely that prey has been disabled in its vicinity.

## Data bibliography

1. Livingstone, P. G., Millard, A. D., Swain, M. T. & Whitworth, D. E. Sequence Read Archive. SRX3143879, SRX3143880, SRX3143934, SRX3143935, SRX3143947, SRX3143951, SRX3143956, SRX3143960 and SRX3143962 (2017).

## Supplementary Data

1971 Supplementary File 1

## Supplementary Data

284 Supplementary File 2
