# Supplementary material for: Transcriptional changes when Myxococcus xanthus preys on Escherichia coli suggest myxobacterial predators are constitutively toxic but regulate their feeding
Source: Microb Genom. 2018 Jan 18;4(2):e000152. doi: 10.1099/mgen.0.000152 (PMC5857379; doi:10.1099/mgen.0.000152)

Multi-dimensional scaling plot of predator gene expression in nutrient medium, starvation medium, and with LIVE and DEAD prey.

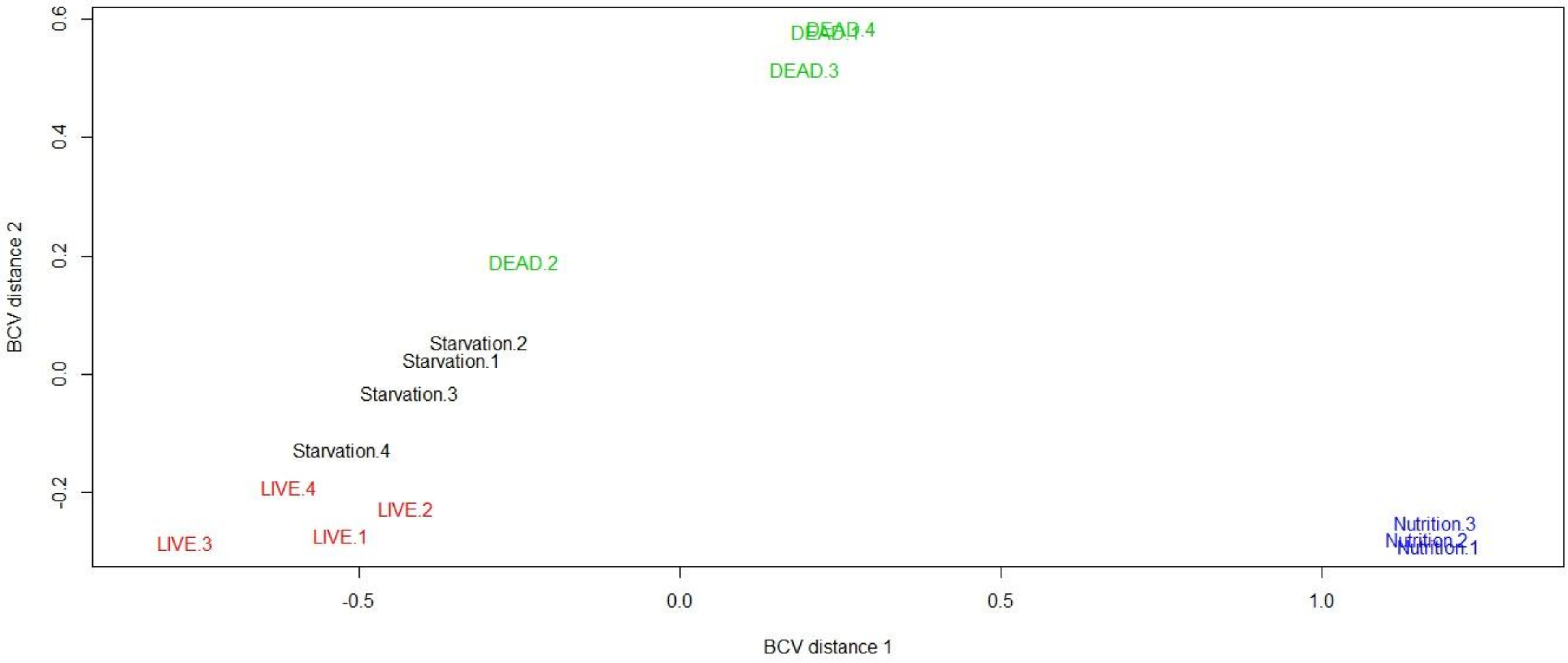

Multi-dimensional scaling plot of prey gene expression, in starvation medium, nutrient medium, and in the presence of predator (LIVE).

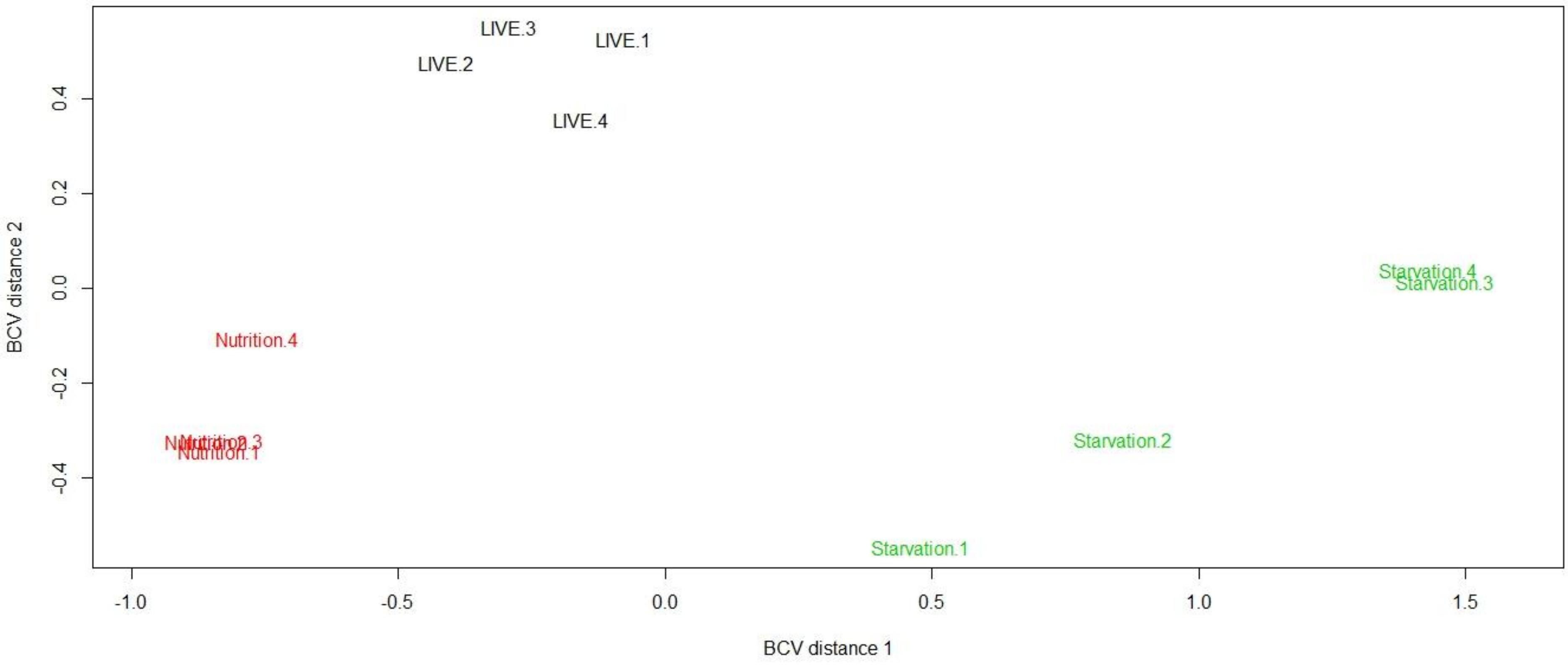

Multi-dimensional scaling plot of prey gene expression, in starvation medium, and in the presence of predator OMVs and culture supernatant.

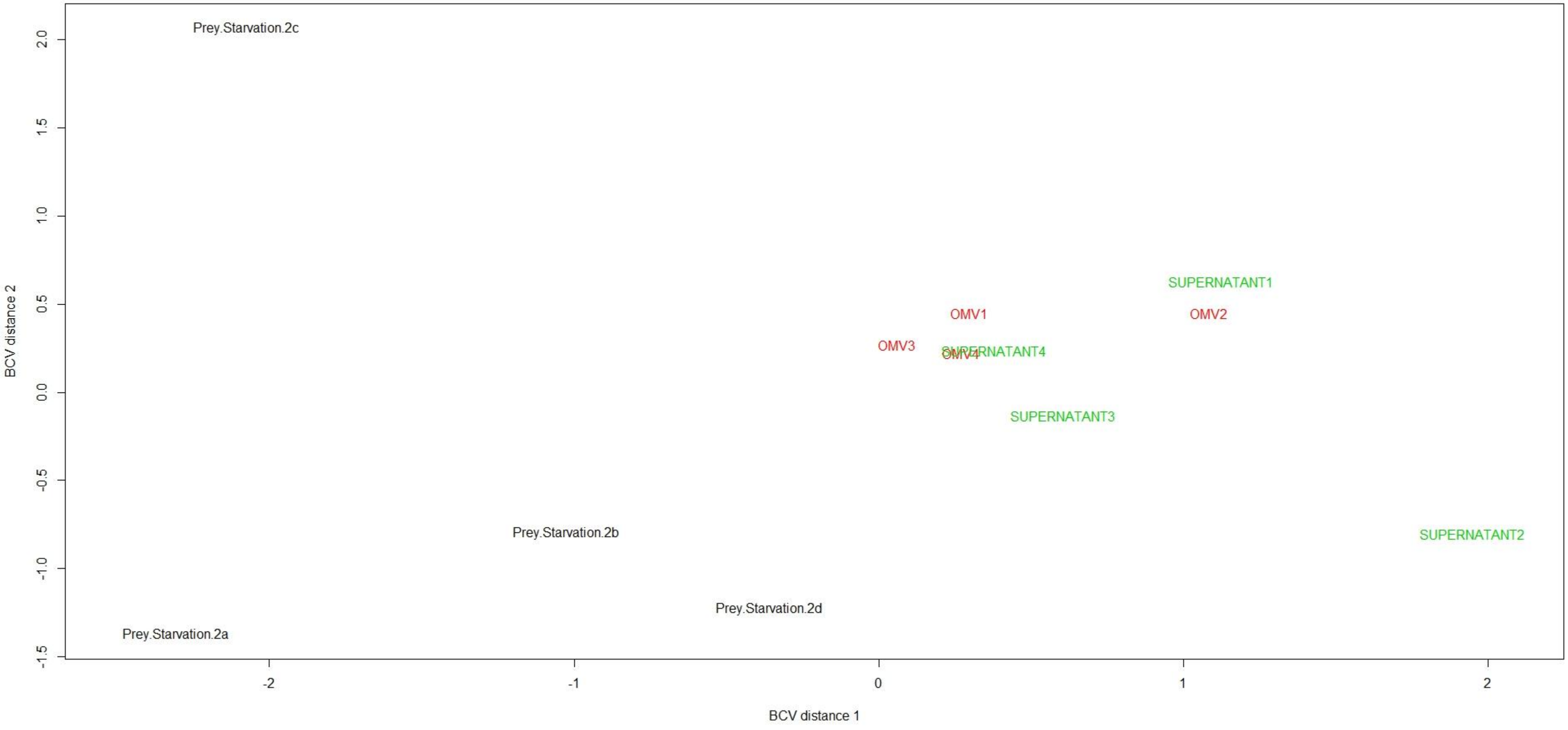

Supplement: Supplementary File 2 [file mgen-4-152-s002.pdf]
